# Age- and BMI-Dependent Psoas and Gluteus Muscle Mass in 27,805 Participants of the Population-Based German National Cohort (NAKO Gesundheitsstudie): A Deep-Learning 3T MRI Study

**DOI:** 10.3390/diagnostics16020205

**Published:** 2026-01-08

**Authors:** Lena Sophie Kiefer, Marius Winter, Sofia Pappa, Marc Fischer, Thomas Küstner, Thierno D. Diallo, Eduardo Calderón, Fabian Bamberg, Konstantin Nikolaou, Bin Yang, Fritz Schick

**Affiliations:** 1 Department of Nuclear Medicine and Clinical Molecular Imaging, University Hospital Tuebingen, Hoppe-Seyler-Straße 3, 72076 Tuebingen, Germany; eduardo.calderon-ochoa@med.uni-tuebingen.de; 2 Department of Diagnostic and Interventional Radiology, Eberhard Karls University of Tuebingen, 72076 Tuebingen, Germany; marius.winter@iss.uni-stuttgart.de (M.W.); pappa.sophia@gmail.com (S.P.); thomas.kuestner@med.uni-tuebingen.de (T.K.); konstantin.nikolaou@med.uni-tuebingen.de (K.N.); fritz.schick@med.uni-tuebingen.de (F.S.); 3 Institute of Signal Processing and System Theory, University of Stuttgart, 70550 Stuttgart, Germany; marc.dc.fischer@gmail.com (M.F.); bin.yang@iss.uni-stuttgart.de (B.Y.); 4 Department of Diagnostic and Interventional Radiology, University Medical Center Freiburg, Faculty of Medicine, University of Freiburg, 79106 Freiburg, Germany; thierno.diallo@uniklinik-freiburg.de (T.D.D.); fabian.bamberg@uniklinik-freiburg.de (F.B.)

**Keywords:** skeletal muscle mass, muscle volume, deep learning, muscle segmentation, German National Cohort

## Abstract

**Background/Objective:** This study aimed to develop and validate an automated deep learning-based model for 3D segmentation and quantification of the psoas major and gluteus muscles at 3T MRI in a large cohort study and to analyze the distribution of findings as well as gender-, age-, and BMI-related differences. **Methods:** The study population consisted of 27,805 participants from the MR imaging subgroup of the population-based, longitudinal German National Cohort study. A deep learning segmentation model was trained, tested, and implemented to automatically quantify psoas major maximum cross-sectional area (CSA_psoas_) and gluteus volume (V_gluteus_) on T1-weighted 3D VIBE DIXON sequences. Associations with gender, age, and BMI were assessed by linear regression. **Results:** The segmentation model demonstrated a high performance, with mean Dice coefficients of 0.92 for the psoas and 0.95 for the gluteus. Males showed higher total CSA_psoas_ (males: 37.92 ± 5.80 cm^2^; females: 24.47 ± 3.65 cm^2^) and higher total V_gluteus_ (males: 3.384 ± 0.528 L; females: 2.386 ± 0.408 L) compared to females. Younger participants aged <30 years showed the highest CSA_psoas_, whereas participants aged 30–59 years showed the highest V_gluteus_. Participants with higher BMI > 25 kg/m^2^ showed higher muscle CSA and volumes compared to subjects with lower BMI < 25 kg/m^2^. V_gluteus_ showed a strong correlation to body weight in both females and males. **Conclusions:** Deep learning-based models provide accurate 3D segmentation and quantification of skeletal muscle compartments from MR images in large cohort studies, thus offering a feasible method for skeletal muscle evaluation. The morphometric size characteristics of the psoas and gluteus muscles are dependent on gender and BMI. Deep learning enables accurate 3D segmentation and quantification of skeletal muscle in large MR imaging cohorts, providing a feasible tool for muscle evaluation. The morphometric characteristics of psoas and gluteus muscles are dependent on gender and BMI.

## 1. Introduction

Human skeletal muscle is one of the largest body compartments and has a fundamental impact on health by enabling physical mobility and playing a key role in both hormone and nutrient metabolism and homeostasis. It may therefore serve as an ideal target for the maintenance and/or improvement of health and its properties (such as muscle mass and structural characteristics) are expected to represent predictors of mobility and autonomy as well as the development and progression of diseases [1,2].

Given its central role in health maintenance, these changes manifest in both hypertrophic changes with adaptive conversion of muscle fibers and involutional changes due to hormonal and inflammatory dysregulation leading to intramuscular lipid and connective tissue infiltration. The main consequences of the latter are not only restricted mobility and physical disability with an increased risk of downfalls, but also negative effects on cardiovascular, metabolic, and neuro-psychologic health with consecutively increased adverse health events and all-cause mortality [1,2,3,4]. Among the various muscle groups, the musculature of the lower trunk and proximal lower limbs has a particularly fundamental impact on the human body due to its complex functional properties and high proportion of total body muscle mass.

The psoas major muscle connects the vertebral column with the lower extremity and is considered to be one of the most significant muscles contributing to posture and statics as well as movement in activities of daily living. Besides its role as the primary flexor of the hip joint, it also stabilizes, flexes, and rotates the lower thoracic and lumbar spine. The gluteal muscles are a group of three muscles (gluteus maximus, medius, and minimus) with the gluteus maximus generally representing the largest muscle in the human body. Besides their function as main pelvic stabilizing muscles, they also provide extension, abduction/adduction and rotation of the hip joint [5,6].

To accurately assess these functionally important muscle groups, advanced imaging techniques are essential. State-of-the-art magnetic resonance imaging (MRI), particularly chemical shift encoding-based water–fat MRI, such as the DIXON-based technique, provides gapless, nearly isotropic 3D image datasets with a high spatial resolution [7,8], thereby enabling non-invasive and highly resolved morphological (e.g., mass) and textural (e.g., biochemical water–fat composition) assessment of skeletal musculature. However, the need for extensive time-consuming manual segmentation—even when performed on a single 2D slice—has prevented the routine evaluation of these measures [9]. The development of automated segmentation models addresses this limitation by offering not only the possibility to assess muscle characteristics in individuals, but also enabling more profound evaluation of the role of different muscle compartments in health and disease within large-scale cohort studies [10,11].

The German National Cohort (NAKO Gesundheitsstudie) represents an ideal platform for implementing such automated approaches. This population-based, longitudinal cohort study comprises approximately 200,000 randomly selected participants, mainly Caucasians (less than 5% non-Caucasians) [9]. The main objective of the German National Cohort is to evaluate the general disease burden and to investigate associations between chronic diseases (e.g., cardio-metabolic disease) and lifestyle and both socio-economic and environmental factors. A subgroup of ∼30,000 participants was enrolled in the MRI sub-study, which included whole-body MRI examinations providing dedicated neurologic, cardiovascular, thoraco-abdominal as well as musculoskeletal image data [10]. With recent advances of deep learning-based, fully automated 3D segmentation models, the processing of these large imaging-related datasets together with the rich non-imaging data offers new perspectives and enables novel insights into normal distribution of muscle characteristics as well as their alterations with aging and disease.

The objectives of this study were twofold: the first was to develop and validate a fully automated, 3D deep learning-based model for segmenting and quantifying the cross-sectional area of psoas major and the volume of gluteus at 3 Tesla MRI derived from the German National Cohort; and the second was to analyze the distribution of muscle sizes and their associations with gender, age and BMI.

## 2. Materials and Methods

### 2.1. German National Cohort (NAKO Gesundheitsstudie)—Study Design and Participant Database

All participants for this study were drawn from the German National Cohort, a population-based, multi-center, longitudinal observational study including approximately 200,000 randomly selected participants from the general German population aged 19 to 74 years. The aims and organization of the NAKO as well as inclusion and exclusion criteria have been described in detail elsewhere [10]. In brief, the objective of this study is to investigate major chronic diseases (such as cardio-metabolic and neurodegenerative diseases, cancer, and musculoskeletal diseases), their pre-clinical phenotypes, risk factors and associated MRI-based imaging biomarkers [10]. All participants underwent a dedicated medical examination including standardized interviews and questionnaires, physical exams and biospecimen collection. A subgroup of approximately 30,000 randomly selected participants underwent whole-body 3 Tesla MRI scanning. Participants were excluded only if imaging data were corrupted or anthropometric data were missing.

The study was approved by the ethics committee and data analysis was approved by the local institutional review boards. All participants provided written informed consent.

### 2.2. MR Image Data Acquisition

Whole-body MR examinations were performed at five imaging centers across Germany using 3 Tesla Magnetom Skyra scanners (Siemens Healthineers, Erlangen, Germany) and a standardized imaging protocol. Participants were scanned in the supine head-first position using an 18-channel body surface coil combined with a 32-channel table-mounted spine matrix coil. The complete imaging protocol and technical specifications have been described in detail previously [12].

Each examination included a T1-weighted 3D dual-echo VIBE DIXON sequence acquired in axial orientation using the following imaging parameters: resolution of 3 mm slice thickness, 1.4 mm by 1.4 mm in-plane resolution, echo times: 1.23 and 2.46 ms, repetition time: 4.36 ms, flip angle: 9°, four scanning positions covered the neck, thorax, abdomen, pelvis and thighs. Water- and fat-selective images were automatically calculated on the MR scanners and compiled into 3D DICOM datasets covering the body trunk and upper thigh. These datasets were converted to NifTI format for further processing.

### 2.3. Manual Muscle Segmentation—Ground Truth Labeling

To generate suitable training data, manual segmentation was performed on a random sample of 20 MR datasets (demographics of the whole study cohort are provided in Table 1, demographics of the training datasets are provided in Table 2) from the first data release of the MR sub-study using the dedicated software 3D Slicer [13]. The segmentations were conducted by an experienced radiologist (>5 years of MRI experience), who was blinded for clinical parameters.

For this purpose, water contrast images from each dataset were imported and displayed primarily in the axial plane. Coronal and sagittal orientations of the water images as well as other contrasts were also used when helpful. The segmentation of a single dataset took approximately 60 min. The resulting manual segmentation masks were then exported in NIfTI format for further processing.

**Figure 1:**
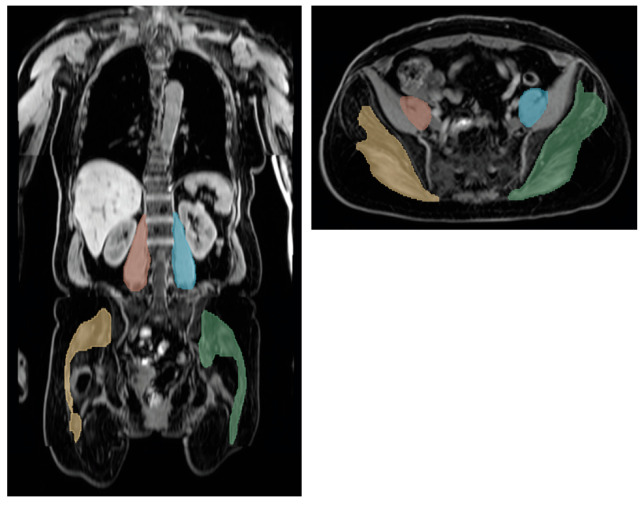
Automatic 3D segmentation of the psoas and the gluteus muscle compartments in a 61-year-old female with a BMI of 22.8 kg/m^2^. Psoas muscle: right side of body in red and left side of body in blue. Gluteus muscles: right side of body in yellow and left side of body in green.

The two muscle compartments analyzed in this study are listed below (Figure 1):

1. The psoas compartment comprising the psoas major and minor muscles, segmented from the muscle origin (at the T12 to L4 vertebral bodies and intervertebral discs) to their distal union with the iliacus muscle in the lesser pelvis.
2. The gluteal muscle compartment comprising the gluteus maximus, medius, and minimus muscles from their origin at the sacrum, ilium, and coccyges to its insertion at the femur as well as the piriformis muscle, which spans from the internal surface of the sacrum to its insertion at the femur.

The resulting manually segmented image volumes were used to train and validate a 3D convolutional network (3D U-Net model: nnU-Net, full-resolution configuration, further description in the following paragraph) to perform the volumetric segmentation of these two muscle compartments on both sides of the body. Thus, the nnU-Net is inherently capable of identifying the above-described muscle boundaries without requiring task-specific modifications to the model for the region of interest.

### 2.4. Automated Muscle Segmentation—Training

To develop the automated segmentation pipeline, the state-of-the-art deep learning framework for biomedical image segmentation, known as nnU-Net, was implemented [14]. This framework is built upon a convolutional encoder–decoder network architecture configured with standard parameters: 1000 epochs, polynomial learning rate decay with initial learning rate of 0.01, stochastic gradient descent optimization with Nesterov momentum and µ = 0.99, and a combined Dice and cross-entropy loss. It provides automated and standardized configurations for data preprocessing, postprocessing, and ensembling, and automatically adapts its configuration to the characteristics of the input data. For the purpose of this study, we used the 3D full-resolution configuration of the nnU-net and trained the model using a 5-fold cross-validation scheme. All four DIXON contrasts were used (in-phase, out-of-phase, fat, and water contrasts) as a 4-channel input. Specifically, a single model was trained to segment both muscle groups—the psoas and the gluteus compartments—on both the left and right sides of the body. Thus, the model output two classes (one per side for each muscle group). A total of *N* = 70 manually segmented and annotated datasets were used for training. All training was conducted on a single dedicated GPU (Nvidia V100, Nvidia, Santa Clara, CA, USA).

### 2.5. Automated Muscle Segmentation—Testing and Implementation

Model-generated segmentations were evaluated using the Dice similarity coefficient (DSC). The quantitative evaluation of the automated segmentation performance was based on quantitative metrics comparing automated and manual segmentations. Mean values were computed across all datasets. Additionally, Bland–Altman plots were used to visualize the agreement between manual and automated muscle segmentations.

**Figure 2:**
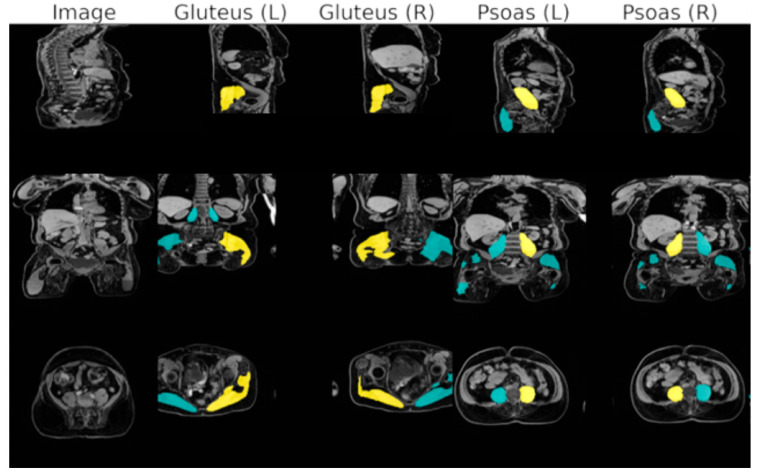
Maximum intensity projection (MIP) images displayed in sagittal, coronal, and axial directions allowing for quick visual quality control of the automatic 3D muscle segmentations in the large German National Cohort.

For quality assurance, a manual consistency check of the predicted segmentation masks was conducted using an overlay plot generated for all 11,687 subjects from the first data release of the MR sub-study (Figure 2). The plot displayed the water contrast of the DIXON sequence and the four muscle compartments (on both the left and right side of the body) along three axes (sagittal, coronal, and axial). Two visualizations were provided: (1) A maximum intensity projection (MIP) of the segmented muscle regions (in the upper rows) and (2) cross-sectional views along the geometric center of the muscle (indicated in yellow, with all the other segmentation groups in turquoise in the lower three rows). This enabled visual detection of segmentation errors, fat–water swaps of the DIXON sequence, and corrupted DICOM header/orientation inconsistencies.

As the psoas muscle compartment could not be assessed in its total volumetric extent (due to its union with the iliacus muscle joining together at the minor trochanter as the iliopsoas muscle), the maximum cross-sectional area of the psoas compartment in cranio-caudal orientation (along the *z*-axis) was extracted (CSA_psoas_) from the volumetric segmentation [15,16,17]. The total volume of the gluteus muscle compartment was extracted (V_gluteus_). Furthermore, the spatial distribution of the CSAs was assessed considering the percentage of total muscle area along the cranio-caudal axis. All data were automatically derived from the generated segmentation masks.

### 2.6. Health Assessment and Anthropometric Data

All participants underwent a standardized health assessment at the study centers, including interviews, questionnaires, physical and laboratory examinations to determine main characteristics, demographics (e.g., age and gender) and important covariates (e.g., body weight).

Anthropometric data such as body weight (in kg) and body height (in m) were measured at the study centers using standardized devices (the medical Body Composition Analyzer 515 and Stadiometer 274, respectively, both from Seca GmbH, Hamburg, Germany). The body mass index (BMI) was calculated as body weight (in kg) divided by body height squared (in m^2^).

### 2.7. Statistical Analysis

Data are generally reported as mean ± standard deviation (SD) for continuous variables and absolute counts with percentages for categorical variables. Furthermore, the 10th, 50th and 90th percentiles were calculated for different age groups and separately for male and female participants.

Intra-reader similarity (IRS) was assessed using the Dice similarity coefficient (DSC) calculated from true-positive (TP), false-positive (FP) and false-negative (FN) annotated image pixels:

$$DSC=\frac{2TP}{2TP+FP+FN}$$

Model training was further evaluated using class-wise metrics derived from the confusion matrix (see Table 3).

The association of skeletal muscle cross-sectional area and volume with anthropometric data (gender, age, and BMI) was assessed using linear regression and Pearson’s correlation coefficient. Statistical significance was set at *p*-value < 0.05. All statistical analyses were conducted in Python 3.11 (Python Software Foundation, Beaverton, OR, USA) and the SciPy.Stats library (scipy.stats, version 1.16.1).

## 3. Results

### 3.1. Study Population

A total of 30,927 datasets were available from the first and second data releases of the German National Cohort MRI sub-study. In total, 3122 datasets were excluded due to corrupted or missing imaging data. Among them, 712 were excluded for image or segmentation errors (see the next paragraph for details). For analyses involving BMI, an additional 334 females and 612 males were excluded due to missing anthropometric data (height or weight). Thus, the final study cohort included 27,805 participants, of whom 44.1% were female and 55.9% were male. The mean age of all participants was 48.2 ± 12.3 years (males: 47.9 ± 12.4 years and females: 48.7 ± 12.2 years, respectively). The mean BMI was 26.4 ± 4.4 kg/m^2^ (males: 26.7 ± 3.8 kg/m^2^; and females: 25.9 ± 5.1 kg/m^2^). The demographic and anthropometric characteristics of the study cohort, as well as the training population, are summarized in Table 1 and Table 2.

### 3.2. Automatic Segmentation Using a DL-Based Segmentation Model

**Figure 3:**
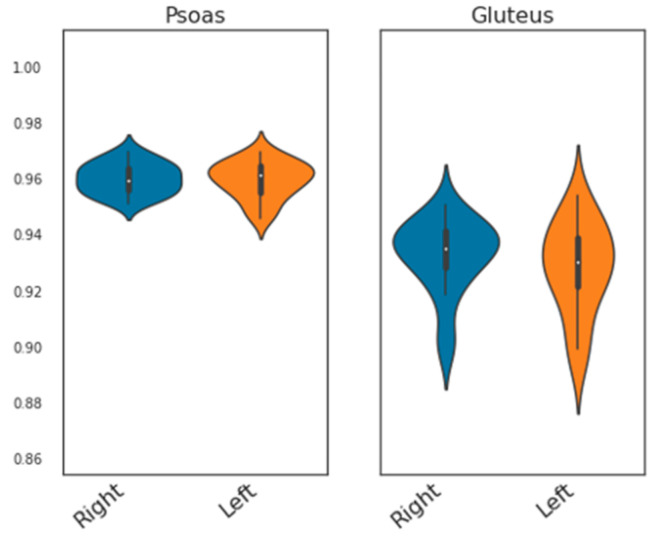
Agreement of manual and automated 3D muscle segmentation. Violin plots comparing Dice Scores of automated segmentation results for the psoas and gluteus compartments for the right (blue) and left (orange) side of the body.

Quantitative evaluation of automated segmentation performance was conducted by comparing automated and manual segmentations in 20 randomly selected datasets using standard metrics. Comprehensive performance results are provided in Table 3. Overall, the performance metrics were indicative of very strong agreement between automated segmentations with manual ground truth. Five-fold cross-validated training of the nnU-Net yielded mean Dice Similarity Coefficients (DSCs) of 0.921 ± 0.016 and 0.925 ± 0.013 for the left and right psoas compartment, and 0.952 ± 0.006 and 0.953 ± 0.005 for the left and right gluteus compartment, respectively. The psoas segmentation scores were tightly centered around the median, whereas gluteus segmentation showed a broader distribution (Figure 3). Bland–Altman plots showed a good agreement and low bias of the manual and automated quantification of CSA_psoas_ and V_gluteus_.

**Figure 4:**
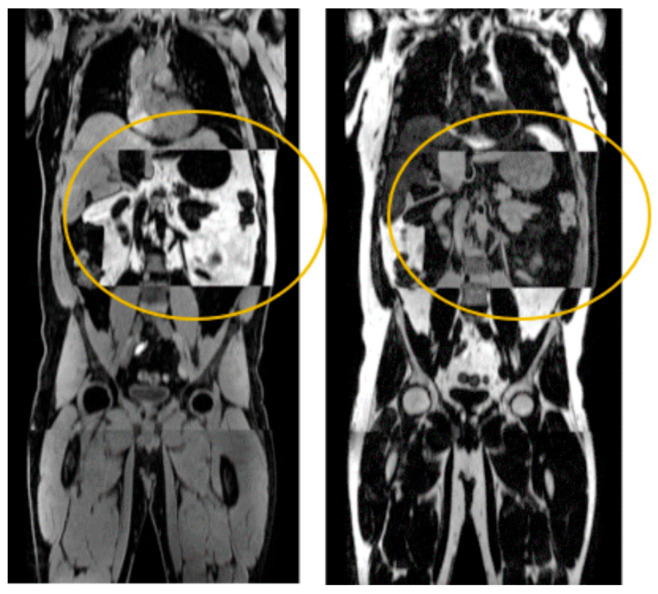
Outlier because of an extended fat–water swap in one of the segments used for composition of coronal water and fat images.

In the first data release of the MR sub-study comprising 11,189 participants, visual inspection identified and excluded 712 datasets (6.4% of the whole population) due to imaging artifacts, such as partial fat–water swaps in the DIXON sequence, affecting the region of interest (Figure 4). Some false positive outliers that were retained, such as cases with inaccurate identification of the distal psoas–iliacus junction. Visual assessment of the remaining 10,477 MR datasets revealed no systematic errors related to gender, age or BMI, indicating overall consistency across the cohort.

### 3.3. Psoas Muscle Cross-Sectional Area and Gluteus Muscle Volume

**Figure 5:**
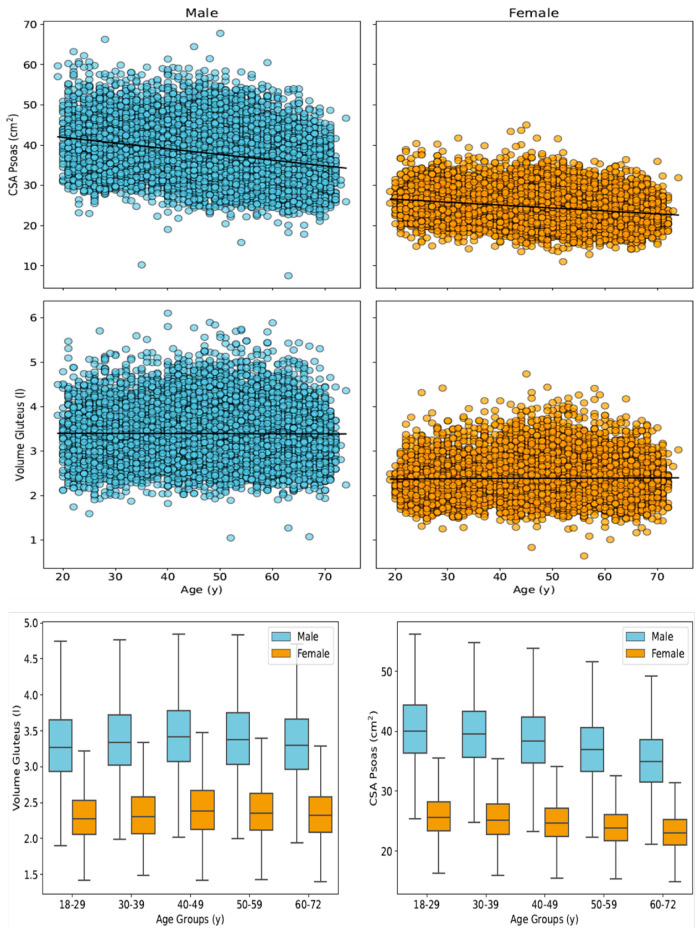
Distribution of muscle sizes (cross-sectional area of psoas and volume of gluteus muscle) for male and female subjects in five different age groups.

**Figure 6:**
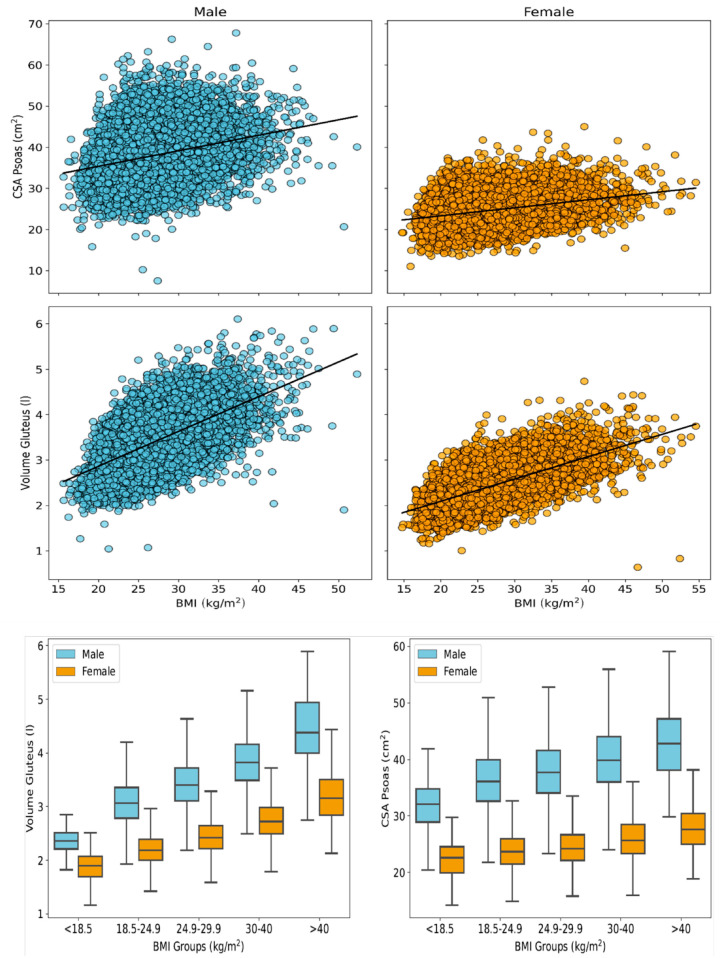
Distribution of muscle sizes (cross-sectional area of psoas and volume of gluteus muscle) for male and female subjects in five different BMI groups.

The results of psoas and gluteus muscle segmentations are presented in Table 1, Figure 1 and Figure 2. Across the full study cohort, female participants exhibited lower skeletal muscle mass than males in all age groups (psoas: 35.5% lower CSA in females compared to males; gluteus: 29.3% lower V in females compared to males). In females, the mean total CSA_psoas_ was 24.47 ± 3.65 cm^2^ (95% CI: 24.41–24.53 cm^2^; left side: 12.34 ± 1.91 cm^2^; and right side: 12.12 ± 1.87 cm^2^). In male participants, the mean CSA_psoas_ was higher, namely 37.92 ± 5.80 cm^2^ (95% CI: 37.83–38.01 cm^2^; left side: 19.14 ± 3.02 cm^2^; and right side: 18.79 ± 2.98 cm^2^). When stratified by age, the highest CSA_psoas_ values were observed in participants aged 18–29 years, while the lowest were seen in those aged 60–72 years (Figure 5) in both males and females. The total gluteus muscle volume (V_gluteus)_ was also lower in females (2.39 ± 0.41 L, 95% CI: 2.38–2.40 L) compared to males (3.38 ± 0.53 L, 95% CI: 3.37–3.39 L). Interestingly, both male and female participants aged 40–49 years showed the highest V_gluteus values,_ exceeding those of younger subjects (<40 years) and older (>50 years) individuals. An increasing BMI was associated with higher CSA_psoas_ and V_gluteus_, reaching a maximum in obese participants with BMI > 40 kg/m^2^ (Figure 6).

### 3.4. Spatial Distribution of Muscle Mass

**Figure 7:**
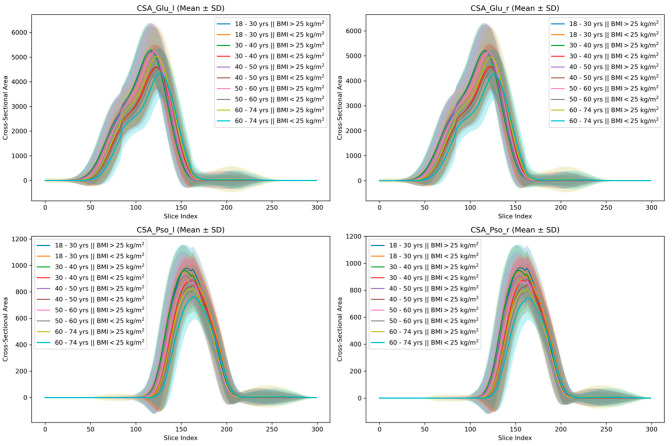
Spatial distribution of psoas and gluteus muscle mass along the cranio-caudal axis according to age and in two different BMI subgroups (lower and higher 25 kg/m^2^) demonstrating geometric scaling across BMI categories. CSA is unadjusted for body height.

Findings on the spatial distribution of muscle mass along the cranio-caudal axis are provided in Figure 7. Age-dependent differences were evident in both normal-weighted and overweight female and male participants. With increasing age, the location of the maximum CSA_psoas_ shifted distally from the upper lumbar spine towards the hip joint region. A similar distal shift in the maximum CSA_psoas_ was also observed within age groups in participants with BMI > 25 kg/m^2^ compared to normal-weighted individuals. Additionally, female participants showed a greater proportion of muscle mass located in the more distal muscle parts (pelvic region) of both the psoas and gluteus compartments.

### 3.5. Correlation Analysis

Correlation results for CSA_psoas_ and V_gluteus_ are provided in Table 4. Both measures demonstrated negligible correlation with age in both female and male participants. V_gluteus_ showed a strong correlation with body weight in both sexes, particularly in females. CSA_psoas_ also showed the strongest correlation with body weight, though the relationship was weak in both sexes. Regarding BMI, a strong correlation was observed between V_gluteus_ and BMI in females and a moderate correlation in males. BMI and CSA_psoas_ were only weakly correlated.

## 4. Discussion

This study trained, tested, and implemented a state-of-the-art deep learning-based automated 3D segmentation (nnU-Net) model for the analysis of the psoas and gluteus muscle compartments in MRI data from 27,805 participants of the first and second data release of the MR sub-study from the German National Cohort. The model achieved robust and accurate segmentation and quantification, thus enabling further analysis of normative distributions and gender-, age-, and BMI-related differences in muscle size characteristics within this large population-based cohort study.

Automated MR segmentation of skeletal muscle compartments (specifically the psoas and gluteus muscle compartments) using a nnU-Net architecture yielded state-of-the-art performance without requiring manual post-processing. Robust segmentation quality was demonstrated by low standard deviations in evaluation metrics derived from 20 randomly selected and manually annotated training datasets from the German National Cohort. Visual inspection of over 10,000 cases confirmed a low outlier rate. In this regard, our model outperformed prior methods, achieving a mean Dice Coefficient (DSC) ≥ 0.92 for both psoas and gluteus muscle compartments, compared to prior studies (e.g., Jung, M. et al., who reported DSC ≥ 0.91 for psoas, autochthonous and abdominal muscles). Previous studies have encountered limitations in accurately delineating skeletal muscle tissue potentially causing inaccurate quantification; for example, by including extra-muscular fat tissue around the spine, bone marrow, and parts of adjacent organs. In contrast, our approach employed standardized segmentation according to anatomical landmarks, improving consistency and minimizing oversegmentation. Given the model’s high confidence scores and manual validation, it can be directly applied to large-scale datasets, such as those in the German National Cohort.

This study confirms and extends results from earlier studies with smaller cohorts such as the KORA study [8]. The high spatial resolution of the MR data of the German National Cohort enabled automated 3D volumetric segmentation and detailed analysis of the spatial distribution of muscle mass along the cranio-caudal axis. The use of a consistent, high-quality imaging protocol and a uniform, large-scale dataset from the NAKO supports a nuanced understanding of how muscle mass varies with anthropometric traits, age, as well as gender.

This study has some limitations. First, only less than 5% of participants in the German National Cohort are non-Caucasians, which limits the generalizability of findings across ethnic groups. This is an important consideration given that skeletal muscle mass and distribution parameters can vary significantly across ethnic groups. For instance, studies have shown that individuals of Asian descent tend to have lower skeletal muscle mass compared to Caucasians, while African Americans often exhibit greater muscle mass and strength, which may affect the interpretation of normative values. Therefore, the normative values presented in this study primarily reflect a Central European/Caucasian population. Given the potential ethnic differences in muscle characteristics, caution is warranted when applying these values to non-Caucasian populations. In particular, the trained models for skeletal muscle mass and distribution may need to undergo transfer learning or re-training to account for the specific anatomical characteristics of Asian or African American populations. This will ensure that the models are generalizable and accurate when applied to diverse ethnic groups [18,19]. Second, this analysis did not include clinical variables associated with diseases (e.g., metabolic or laboratory parameters) or laboratory biomarkers; it was limited to anthropometric data and image-derived muscle mass. Also, absolute muscle CSA is inherently influenced by body size and height-related geometric scaling; therefore, normalized indices such as CSA/height^2^ may be preferable for cross-sectional comparisons across individuals of different stature, although this was not the primary focus of the present analysis. Third, due to the spatial resolution of 1.4 × 1.4 × 3 mm, partial volume effects at muscle boundaries may lead to underestimation of small muscle bundles, particularly in women and older individuals with reduced muscle mass, and may adversely affect Dice similarity metrics despite small absolute segmentation errors.

In conclusion, this study demonstrates that deep learning-based automated 3D segmentation of the psoas and gluteus muscle compartments enables fully automated and reliable quantification of skeletal muscle mass and distribution along the cranio-caudal axis from whole-body MRI. This method reveals new insights into the age-, gender-, and BMI-related distribution of skeletal muscle in a large, representative cohort.

In the future studies using 3D MRI from large cohorts will facilitate in-depth investigation of the associations between skeletal muscle mass and composition and cardiometabolic and musculoskeletal diseases, offering more clinically actionable perspectives.

## Figures and Tables

**Table 1:** Demographic data of the *N* = 27,805 study cohort.

	Female					Male				
	Mean ± SD	Range	Percentiles			Mean ± SD	Range	Percentiles		
			10th	50th	90th			10th	50th	90th
*N* (total number and in %)	12,250 (44.1%)					15,555 (55.9%)				
Age (in years)	48.7 ± 12.2	19.0–74.0	29.0	49.0	64.0	47.9 ± 12.4	19.0–74.0	29.0	49.0	64.0
Body weight (in kg)	70.8 ± 13.9	37.3–122.3	55.4	68.2	89.7	85.6 ± 12.9	45.8–122.4	69.9	84.4	103.4
Body height (in m)	165.6 ± 6.5	145.0–190.4	157.2	165.4	174.1	179.1 ± 6.9	150.7–201.2	170.3	179.0	188.0
BMI (in kg/m^2^)	25.9 ± 5.1	14.7–49.8	20.4	24.8	33.0	26.7 ± 3.8	15.6–45.3	22.2	26.3	31.8
CSA_psoas_ total (in cm^2^)	24.47 ± 3.65	5.22–45.01	20.07	24.19	29.21	37.92 ± 5.80	15.80–58.20	30.77	37.61	45.62
CSA_psoas_ left (in cm^2^)	12.34 ± 1.91	2.33–24.34	10.05	12.20	14.80	19.14 ± 3.02	3.46–29.72	15.42	18.96	23.12
CSA_psoas_ right (in cm^2^)	12.12 ± 1.87	2.16–22.43	9.89	12.00	14.55	18.79 ± 2.98	3.36–29.12	15.15	18.63	22.74
V_gluteus_ total (in L)	2.39 ± 0.41	0.83–4.737	1.92	2.34	2.90	3.38 ± 0.53	1.04–5.04	2.72	3.36	4.09
V_gluteus_ left (in L)	1.20 ± 0.21	0.40–2.33	0.96	1.18	1.47	1.70 ± 0.27	0.50–2.54	1.36	1.69	2.06
V_gluteus_ right (in L)	1.19 ± 0.20	0.423–2.46	0.95	1.16	1.45	1.68 ± 0.27	0.54–2.52	1.35	1.67	2.04

**Table 2:** Demographic data of *N* = 59 training datasets.

	Females		Males	
*N* (total number)	30		29	
	Mean ± SD	Range	Mean ± SD	Range
Age (in years)	52.7 ± 9.7	33.0–71.0	50.1 ± 9.1	29.0–68.0
Body weight (in kg)	69.4 ± 11.5	49.5–97.2	89.2 ± 15.3	58.1–114.1
Body height (in m)	163.7 ± 5.1	154.2–176.0	179.6 ± 7.4	167.0–193.5
BMI (in kg/m^2^)	25.9 ± 4.2	17.8–36.4	27.6 ± 4.4	18.0–36.6

**Table 3:** Model performance metrics. Quantitative agreement between automated and manual segmentation represented by different performance metrics for the two muscle compartments on both sides of the body. Data are presented as average values over the 20 datasets drawn from the cross-validation process.

	Psoas Left	Psoas Right	Gluteus Left	Gluteus Right
Accuracy	0.99975 ± 0.00007	0.99977 ± 0.00007	0.99899 ± 0.00023	0.99901 ± 0.00023
Dice	0.92100 ± 0.01590	0.92533 ± 0.01282	0.95247 ± 0.00640	0.95303 ± 0.00527
False discovery rate	0.06969 ± 0.02179	0.06634 ± 0.02669	0.04126 ± 0.01022	0.04186 ± 0.00894
False-negative rate	0.08677 ± 0.03551	0.08131 ± 0.03210	0.05353 ± 0.01258	0.05194 ± 0.00764
False-omission rate	0.00014 ± 0.00008	0.00012 ± 0.00006	0.00058 ± 0.00018	0.00056 ± 0.00016
False-positive rate	0.00011 ± 0.00005	0.00010 ± 0.00006	0.00044 ± 0.00013	0.00044 ± 0.00012
Jaccard	0.85397 ± 0.02711	0.86129 ± 0.02189	0.90933 ± 0.01162	0.91032 ± 0.00962
Negative predictive value	0.99986 ± 0.00008	0.99988 ± 0.00006	0.99942 ± 0.00018	0.99944 ± 0.00016
Precision	0.93031 ± 0.02179	0.93366 ± 0.02669	0.95874 ± 0.01022	0.95814 ± 0.00894
Recall	0.91323 ± 0.03551	0.91869 ± 0.03210	0.94647 ± 0.01258	0.94806 ± 0.00764

**Table 4:** Pearson correlations of muscle mass with anthropometric data.

	Gluteus Total		Psoas Total	
	Females	Males	Females	Males
Age	0.02	−0.002	−0.24 **	−0.29 **
Body height	0.39 **	0.47 **	0.33 **	0.29 **
Body weight	0.78 **	0.75 **	0.39 **	0.37 **
BMI	0.62 **	0.54 **	0.26 **	0.24 **

** *p* < 0.001.

## Data Availability

The original contributions presented in this study are included in the article. Further inquiries can be directed to the corresponding author.

## Abbreviations

BMI	Body mass index.
cm	Centimeters.
CSA	Cross-sectional area.
DICOM	Digital Imaging and Communications in Medicine.
DSC	Dice Score Coefficient.
FP	False positive.
FN	False negative.
GNC	German National Cohort.
IRS	Intra-reader similarity.
kg	Kilograms.
L4	Fourth lumbar vertebra.
m^2^	Square meters.
mm	Millimeters.
MR	Magnetic resonance.
MRI	Magnetic resonance imaging.
ms	Milliseconds.
NAKO	Nationale Kohorte.
T	Tesla.
T12	Twelfth thoracic vertebra.
TP	True positive.
V	Volume.
VIBE DIXON	Volumetric Interpolated Breath-hold Examination.
